# QRS Morphology Shift Following Catheter Ablation of Idiopathic Outflow Tract Ventricular Tachycardia

**DOI:** 10.19102/icrm.2020.111202

**Published:** 2020-12-15

**Authors:** Konstantinos P. Letsas, Stelios Dragasis, Athanasia Megarisiotou, Panagiotis Mililis, George Bazoukis, Athanasios Saplaouras, Antonios Sideris, Michael Efremidis

**Affiliations:** ^1^Arrhythmia Unit, Laboratory of Cardiac Electrophysiology, Evangelismos General Hospital of Athens, Athens, Greece

**Keywords:** Ablation, outflow tract, ventricular tachycardia

## Abstract

A 42-year-old patient without structural heart disease was referred for catheter ablation of salvos of outflow tract ventricular tachycardia (VT). Activation mapping of the clinical VT (VT1) revealed the earliest ventricular activation site at the right ventricular outflow tract (RVOT). Catheter ablation at this site led to a slight QRS shift of the VT morphology (VT2). Activation mapping of VT2 established the site of origin at the commissure between the right (RCC) and left (LCC) coronary cusps. This case is indicative of the presence of myocardial fibers displaying preferential conduction properties from the RCC–LCC commissure to a breakout site at the RVOT.

## Case presentation

**Figure 1:**
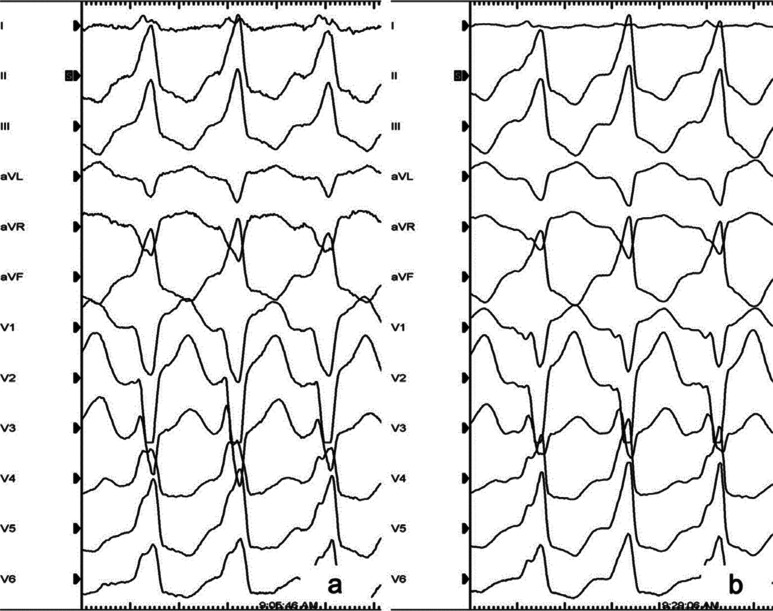
**A:** ECG of the clinical VT (VT1). **B:** ECG of the VT following catheter ablation at the RVOT (VT2). The most prominent change was seen in lead V1, where the rS pattern shifted to a QS pattern with notching on the downward deflection, which is suggestive of an RCC–LCC commissure SOO.

A 42-year-old patient was referred for catheter ablation of symptomatic salvos of idiopathic ventricular tachycardia (VT). Structural heart disease was excluded by means of transthoracic echocardiography, cardiac magnetic resonance imaging, and exercise stress testing. The electrocardiogram (ECG) during VT displayed a left bundle branch block pattern (LBBB) with an inferior axis, R-wave in lead I, and precordial transition in lead V4 **(Figure 1A)**. The QRS morphology was suggestive of a right ventricular outflow tract (RVOT) site of origin (SOO). An electrophysiologic study was performed in a fasting state without sedation. Antiarrhythmic drugs (β-blocker) were stopped at least five half-times before the procedure. High-density activation mapping of the clinical VT (VT1) was performed using a three-dimensional nonfluoroscopic mapping system (CARTO^®^ 3; Biosense Webster, Diamond Bar, CA, USA) via a multipolar catheter (DecaNav catheter, 2-8-2 interelectrode spacing; Biosense Webster, Diamond Bar, CA, USA). A contact force-sensing catheter (SmartTouch™; Biosense Webster, Diamond Bar, CA, USA) was used for validation of the earliest activation site and ablation (contact force up to 8 g).

**Figure 2:**
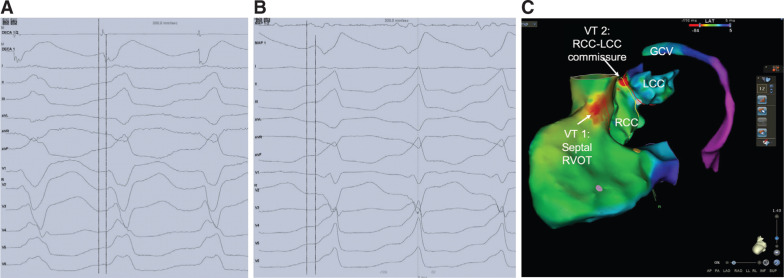
**A:** Intracardiac bipolar (DECA 1–2) and unipolar (DECA 1) electrograms at the earliest activation site of VT1. The unipolar signal displayed a small initial r-wave with a steep negative dV/Dt. **B:** Intracardiac bipolar (MAP 1–2) and unipolar (MAP 1) electrograms at the earliest activation site of VT2. The unipolar signal exhibited an rS pattern with a less steep negative configuration. Of note, very low voltage and fragmented bipolar signals were recorded at the successful ablation site of VT2. **C:** Activation mapping showing the earliest activation site of VT1 at the RVOT and of VT2 at the RCC–LCC commissure.

Activation mapping of the RVOT revealed the earliest activation site at the posterior septum (−23 ms) **(Figures 2A and 2C)**. The unipolar signal at this site displayed a small initial r-wave with a steep negative dV/dT. Radiofrequency (RF) energy delivery (25–30 W, 43°C) at this site led to a slight QRS shift of the VT morphology. The ECG morphology of the second VT (VT2) was characterized by LBBB with an inferior axis, a lower-amplitude R-wave in lead I, and a precordial transition in lead V4 **(Figure 1B)**. Of note, the most prominent change was seen in lead V1, where the rS pattern shifted to a QS pattern with notching on the downward deflection, which is suggestive of a right coronary cusp (RCC)–left coronary cusp (LCC) commissure SOO. Following the change in QRS morphology, an additional attempt of RF delivery was performed at this site but failed to suppress the arrhythmia. Meticulous activation mapping of the VT2 was subsequently performed at the RVOT, the left ventricular outflow tract (LVOT) including the coronary cusps, and the coronary venous system [great cardiac vein (GCV)]. The new activation mapping attempt revealed the earliest activation site at the RCC–LCC commissure (−25 ms) **(Figures 2B and 2C)**. Catheter ablation (20–30 W, 43°C) at this site led to suppression of the arrhythmia. Of note, very low voltage and fragmented bipolar signals were recorded at the successful ablation site **(Figure 2B)**. On the contrary, the unipolar signal exhibited an initial r-wave with a less steep negative dV/dT as compared with during VT1. VT2 was not inducible by atrial or ventricular stimulation with or without isoproterenol infusion 30 minutes after ablation. The patient remained free from arrhythmias six months after the procedure.

## Discussion

This case report is suggestive of the presence of myocardial fibers displaying preferential conduction properties from the RCC–LCC commissure to a breakout site at the RVOT.

Tada et al. initially investigated the prevalence and characteristics of idiopathic outflow-tract ventricular arrhythmias (VAs) with altered QRS morphology following RF catheter ablation.^1^ Of 202 patients with monomorphic VT or premature ventricular contractions (PVCs) originating from the outflow tract, six patients (3%) showed changes in QRS morphology following RF energy delivery, requiring an additional RF application at a different site, which was often the LCC. Yamada et al. reported that VAs originating from the coronary cusps display preferential conduction to the RVOT and an insulated myocardial fiber across the ventricular outflow septum may exist in these cases.^2^ Especially, in 25% of patients with VAs of a coronary cusp origin, pacemapping from the RVOT yielded a closer match of the QRS morphology than pacing from the coronary cusp. In these cases, pacemapping identified the preferential breakout site.^2^ In the study by Shirai et al., a QRS morphology shift following catheter ablation at the earliest activation site was observed in 4% of cases of idiopathic outflow-tract VAs. The shifted VT or PVC demonstrated a distinct ECG QRS pattern supporting the notion that different preferential QRS exit sites within the outflow tract were operative in these cases. After remapping, the earliest activation site of the second VT or PVC moved to a different anatomical structure adjacent to the initial ablation site in 65% of cases, which was most frequently the LVOT region (coronary cusps).

The complexity of the myocardial network around the outflow tract with the potential for preferential/alternative conduction and multiple exiting sites has been suggested to explain subtle variations in the QRS morphology of idiopathic outflow-tract VAs following RF ablation.^2,3^ An intramural SOO with preferential conduction channels to specific sites of the outflow tract (ie, RVOT, coronary cusps, coronary venous system) may explain this phenomenon.^3^ The presence of preferential conduction can be unmasked by ablation at the first earliest activation site when the first preferential exit of the VT/PVC is eliminated.^3^ Unipolar signals displaying an rS pattern at both the earliest activation sites, as seen in our case, favors the presence of an intramural SOO.

In conclusion, a QRS morphology shift following catheter ablation of idiopathic outflow-tract VAs is not an uncommon phenomenon. Meticulous activation mapping usually reveals the true SOO in an adjacent site, which is more commonly the coronary cusps.
